# Cardiovascular health trajectories and subsequent cardiovascular disease and mortality: The multi-ethnic study of atherosclerosis (MESA)

**DOI:** 10.1016/j.ajpc.2022.100448

**Published:** 2022-12-09

**Authors:** Qicong Sheng, Jie Ding, Yumin Gao, Reshmi JS Patel, Wendy S Post, Seth S Martin

**Affiliations:** a Johns Hopkins University School of Medicine, Baltimore, MD, USA; b Krieger School of Arts and Sciences, Johns Hopkins University, Baltimore, MD, USA; c Ciccarone Center for the Prevention of Cardiovascular Disease, Division of Cardiology, Department of Medicine, Johns Hopkins University School of Medicine, Baltimore, MD, USA

**Keywords:** Cardiovascular health, Longitudinal trajectories

## Abstract

**Objective:**

Longitudinal trajectories of cardiovascular health (CVH) may reflect vascular risk burden due to prolonged cumulative exposure to non-ideal CVH levels. Identifying individuals who have a higher risk CVH trajectory may facilitate treatment, screening, and prevention. We aimed to characterize 10-year trajectories of CVH and examine the associations between CVH trajectories and subsequent cardiovascular disease (CVD) and mortality.

**Methods:**

We analyzed 3674 MESA participants who completed four exams and remained CVD-free from 2000 to 2011. A 12-point CVH score was calculated based on physical activity, smoking status, body mass index, cholesterol, blood pressure, and glucose. Ideal CVH was defined as a score ≥ 9. Group-based trajectory modeling was used to identify trajectories of ideal CVH. Cox models were used to examine the association of CVH trajectories with incident CVD and death from 2011 to 2018, adjusting for age, sex, race/ethnicity, income, education, and marital status.

**Results:**

Three trajectories were identified based on the probability of achieving ideal CVH: high (*n* = 1251), medium (*n* = 760), and persistently low (*n* = 1663). Almost half (45.3%) of the participants had a persistently low trajectory. During a median of 7.7 years follow-up, 392 incident CVD events and 459 deaths occurred. Compared with the high CVH group, participants in the persistently low CVH trajectory group had elevated risks for CVD (adjusted hazard ratios 1.49, 95% confidence interval 1.15–1.93) and mortality (1.34, 1.06–1.70), and participants in the medium group had moderate risks for CVD (1.17, 0.86–1.59) and mortality (1.15, 0.87–1.53) (p-value for trend 0.002 for CVD, 0.014 for mortality).

**Conclusion:**

Persistently nonideal CVH is a common trajectory. Targeted prevention programs might benefit individuals with persistently nonideal CVH given their elevated risk of subsequent CVD and mortality.

## 1 Introduction

Cardiovascular disease (CVD) is the leading cause of death for the United States population. In 2010, the American Heart Association (AHA) defined seven categories of ideal cardiovascular health (CVH), with a goal of improving CVH by 20% and reducing CVD mortality by 20% in the following decade [1]. These categories of ideal CVH include four behavioral measures (no smoking, healthy diet, being physically active, and maintaining a normal weight) and three biometric measures (blood cholesterol, blood pressure, and blood glucose) [1].

Monitoring the progress of this composite CVH metric toward improved heart health is an essential part of attaining the strategic 2020 goals in primordial prevention, but information on the changes of the composite CVH metric over time in the primary CVD prevention is limited. Previous population-based epidemiology studies have primarily focused on a single CVH assessment or a change in CVH categories based on only two occasions [2,3]. These measures are subject to either random variation or regression to the mean [4]. Very few studies have examined long-term ideal CVH trajectories, which reflect changes in an individual's ideal CVH categories over time by capturing multiple aspects of lifetime patterns including starting points, slope, and cumulative exposure [5], [6], [7]. Furthermore, understanding how variations in ideal CVH contribute to subsequent risk of CVD could help facilitate targeted cardiovascular prevention programs for high-risk populations.

We thus aimed to characterize the ideal CVH trajectories using four assessments over ten years in a multi-ethnic population that is more diverse than prior studies. We then investigated the associations of these trajectories with the incidence of CVD events and mortality. We hypothesized that participants who did not maintain ideal CVH over the assessment period would have a higher risk of developing CVD and death. Particularly, those with a persistently low level of ideal CVH would have the highest risk.

## 2 Methods

### 2.1 Study sample

The Multi-Ethnic Study of Atherosclerosis (MESA) is a prospective cohort study that began in 2000. A total of 6814 participants aged between 45 and 84 years were recruited from July 2000 to September 2002 at six field centers (Baltimore, MD; Chicago, IL; Forsyth County, NC; Los Angeles, CA; New York, NY; and St Paul, MN). Of all study participants, 27.7% were African American, 11.8% were Chinese American, 22.0% were Hispanic, and 38.5% were white. The participants, all free of clinical CVD at baseline, were invited to complete subsequent exams and questionnaires. The study protocol was approved by the institutional review boards at all field centers, and informed consent was provided by all MESA participants. Further study design details have been previously published [8].

**Fig. 1:**
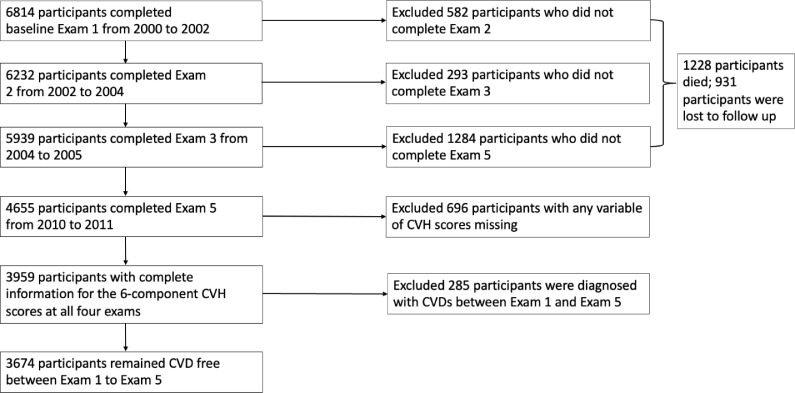
Exclusion and inclusion criteria for the study sample.

Our present study included 3674 MESA participants who completed Exam 1, Exam 2, Exam 3, Exam 5 from 2000 to 2011 and remained CVD-free during this period. We excluded participants with incomplete CVH information at any exam. The exclusion and inclusion criteria are described in Fig. 1. Compared with those included (*n* = 3674) in the final analysis, participants who were excluded (*n* = 3140) were generally older, less likely to be white, less likely to be married, and had lower education and income (Supplementary Table 1).

### 2.2 Measurement of cardiovascular health (CVH)

Six CVH metrics (physical activity, smoking, BMI, blood cholesterol, blood pressure, and blood glucose) were assessed at Exam 1 (2000–2002), Exam 2 (2002–2004), Exam 3 (2004–2005), Exam 5 (2010–2011). Exam 4 was not included due to a lack of physical activity data. Diet quality, also a component of the AHA-defined CVH score, was not used in the CVH score calculation in this analysis because diet information was not available at Exam 2 and Exam 3. A previously-defined point-score system of CVH was used to assign 0 points for poor, 1 point for intermediate, and 2 points for ideal levels of each metric [9]. Details of the CVH metrics are described in Supplementary Table 2. Modifications of CVH metrics in MESA were made with previously described methods [10]. Specifically, physical activity was measured with a questionnaire modified from the Cross-Cultural Activity Participation Study [11]. Scoring of physical activity was based on weekly time spent on moderate and vigorous activities including walking exercise, dance, individual activities, conditioning, and sports. Smoking history was assessed with questionnaires. Scoring of smoking was based on whether participants were currently smoking, previously smoking and quit within the past 12 months, previously smoking and quit more than 12 months ago, or had never smoked. BMI was calculated as weight (kg) divided by height (meters) squared which were measured at each exam. Blood pressure was the average of the second and third measurements taken after participant had rested for 5 min in a seated position. Blood cholesterol and blood glucose were measured from fasting blood samples [8]. Medications that were used to control blood pressure, cholesterol, and glucose were also recorded. With scores of six metrics combined for a total of 12 points, ideal CVH was defined as having a CVH score of 9 or above (this definition is consistent with previous MESA literature which has defined ideal CVH as having a score of 11 or above, out of 14 points with seven metrics [10]).

We created a binary indicator for achieving ideal CVH at each exam and then modeled the four binary indicators as a longitudinal response in a logistic group-based trajectory model (GBTM) [7] to generate different groups of CVH trajectories. GBTM was conducted in a Stata plugin program (Stata Proj Traj). In a GBTM model, several regression models are estimated at the same time, which include a multinomial logistic model calculating the probability of membership in each group, and logistic models calculating the probability of achieving ideal CVH over time as a smooth function of time [12]. Model fit was assessed using the Bayesian Information Criterion, as recommended in the literature, and the number of participants being assigned in each trajectory (>5% of the overall population) [13]. The estimation procedure has been shown to be better in identifying the underlying longitudinal trajectories [14]. Each participant was assigned to the trajectory group for which the individual had the highest posterior probability of membership.

### 2.3 CVD events and death

After the CVH assessment period, beginning after Exam 5, participants were followed for two primary outcomes: CVD and all-cause mortality. CVD events and mortality were identified during participant follow-up every 9 to 12 months and were verified with medical records or interviews [8]. For the purpose of this study, all CVD events were classified as myocardial infarction, resuscitated cardiac arrest, definite angina, probable angina followed by coronary revascularization, stroke, definite or probable heart failure, and cardiovascular death (secondary to stroke, coronary heart disease [CHD], other atherosclerotic death, or other CVD death). For definite or probable heart failure, participants were required to have symptoms and/or signs such as shortness of breath or edema, and a diagnosis of heart failure made by a physician and medical treatment for heart failure. Definite heart failure further required one or more additional objective criteria, such as pulmonary edema/congestion by chest X‐ray, dilated ventricle or poor left ventricular function by echocardiography or ventriculography, or evidence of left ventricular diastolic dysfunction [15]. CHD was defined as myocardial infarction, resuscitated cardiac arrest, definite angina, probable angina if followed by revascularization, and CHD death. Time until event was recorded as days since the participant's Exam 5 date. Each participant was followed until the first record of CVD diagnosis, death, loss to follow-up, or the administrative censoring date (December 31, 2018).

### 2.4 Covariates

Socio-demographic variables were collected by questionnaires at Exam 1 and included the following: sex, race/ethnicity (African American, Chinese American, Hispanic, white), age, education (<high school or high school graduate, some college or college graduate, >college graduate), marital status (married, not married), and income (< $40,000, ≥$40,000 per year). Age, marital status and income information were updated by questionnaires at Exam 5.

### 2.5 Statistical analysis

Baseline characteristics were compared among ideal CVH trajectory categories using analysis of variance (ANOVA) for continuous variables and chi-squared tests for categorical variables. Cox proportional hazards models were fit to obtain the estimated hazard ratios (HRs) and 95% confidence intervals (CIs) for the association of the ideal CVH trajectory subgroup membership (as a categorical variable) with the outcomes of incident CVD and death as well as the composite outcome of CVD and death after the fifth examination cycle. Sub-analyses on CHD, stroke, and heart failure outcomes were also conducted separately. Models were adjusted for age, sex, race/ethnicity, income, education, and marital status. The proportionality assumption was tested using Schoenfeld residuals and was met for all variables. Interactions between categorical ideal CVH trajectory groups and other covariates were assessed by the inclusion of cross-product terms in the fully adjusted models. In sensitivity analysis, we used inverse probability weights (IPWs) to account for selective dropouts or non-random censoring due to loss to follow-up or dropouts (Supplement). All tests were two-tailed, and a two-sided p-value <0.05 indicated statistical significance. All statistical analyses were performed using Stata (StataCorp LP, College Station, TX) software version 15.1 and R version 4.1.2.

## 3 Results

**Fig. 2:**
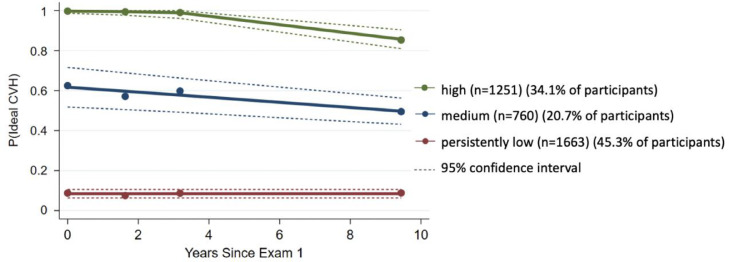
Ideal CVH trajectories from Exam 1 to Exam 5.

Baseline characteristics of the study population at the last exam prior to follow up (Exam 5, 2010–2011) are presented in Table 1. Of the 3674 participants, 53.9% were women and the mean age at baseline was 69.5 years (standard deviation 9.4) (Table 1). Achieving ideal CVH at each exam was best characterized as three latent trajectory groups. The models specifying two, four, or five underlying trajectory groups had lower Bayesian information criterion (BIC) values suggesting a poorer model fit (Supplementary Table 3). Based on the level of achieving ideal CVH for each group, the three latent trajectory groups were labeled “high”, “medium”, and “persistently low” (Fig. 2). A total of 45.3% (1663/3674) of study participants had a persistently low probability of achieving ideal CVH (persistently low trajectory); 20.7% (760/3674) had a medium level of probability of achieving ideal CVH with a slight decline later in time (medium trajectory); the remaining 34.1% (1251/3674) had a high level of probability of achieving ideal CVH with a slight decline later in time (high trajectory). Compared with the persistently low trajectory, participants in the high trajectory were younger, more likely to be men, white, and married, and had higher levels of education and income (Table 1). The distribution of CVH scores in the three trajectory groups at each examination is summarized in Supplementary Figure 1.

**Table 1:** Demographic characteristics of participants at Exam 5 (2010–2011) by their CVH trajectories.

	Total (*n* = 3674) (%)	High (*n* = 1251) (%)	Medium (*n* = 760) (%)	Persistently low (*n* = 1663) (%)	P-value
**Age (mean [sd])**	69.5 [9.4]	68.5 [9.6]	69.6 [9.5]	70.2 [9.2]	< 0.001
**Sex**					
Women	53.9	52.3	51.3	56.3	0.01
**Race/Ethnicity**					
White	40.5	51.2	37.0	34.1	< 0.001
Chinese-American	12.8	18.1	16.1	7.3	
African-American	25.2	15.2	25.7	32.5	
Hispanic	21.5	15.5	21.3	26.0	
**Education**					
≤ High-school graduate	30.8	19.3	29.6	40.1	< 0.001
Some college or college graduate	47.8	48.9	48.8	46.5	
> College graduate	21.4	31.8	21.6	13.4	
**Marital Status (67 missing)**					
Married/Living as married	59.3	65.3	63.8	55.1	< 0.001
**Income (103 missing)**					
< $40,000	43.2	33.6	45.5	52.2	< 0.001
≥ $40,000	56.8	66.4	54.5	47.8

### 3.1 Trends in individual CVH components by ideal CVH trajectory groups

**Fig. 3:**
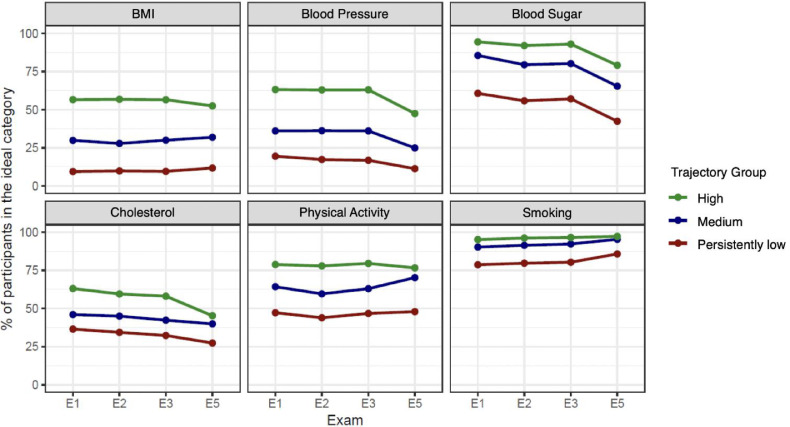
Trends in individual CVH components by ideal CVH trajectory groups (y-axis:% of participants with ideal status of the CVH component; x-axis: Exam).

When examining the trends of each component of the CVH metrics according to ideal CVH subgroups, the proportions of participants with an ideal status of blood pressure, blood glucose, and cholesterol levels decreased from Exam 1 to Exam 5, with the highest decrease observed in ideal blood glucose levels. In contrast, there was an upward trend in ideal smoking status. Ideal levels of BMI and physical activities remained relatively constant. The patterns in the three trajectory subgroups were similar, with the lowest proportions of participants with ideal status in the persistently low trajectory group (Fig. 3, Supplementary Table 4).

### 3.2 CVH trajectories and risk for CVD and all-cause mortality

During a median follow-up of 7.7 years (interquartile range 7.2–8.1), a total of 687 individuals died or had CVD (459 participants died, 392 had CVD, and 164 had CVD and subsequent death). Compared with those in the high CVH category, participants in the persistently low group had a greater risk for subsequent CVD (HR 1.49, 95% CI 1.15–1.93), mortality (1.34, 1.06–1.70), and CVD/mortality as a composite variable (1.39, 1.15–1.69) in multivariable-adjusted models. Participants in the medium group has a moderate risk for CVD (1.17, 0.86–1.59), mortality (1.15, 0.87–1.53), and CVD/mortality as a composite variable (1.12, 0.89–1.41). P-values for trend are 0.002 for CVD, 0.014 for mortality, and <0.001 for CVD/mortality as a composite variable (Table 2). In our sub-analyses on CHD, stroke, heart failure outcomes, the participants in the persistently low group demonstrated an elevated risk for subsequent CHD (1.87, 1.28–2.71) and heart failure (2.1, 1.25–3.52) but not for stroke (0.85, 0.54–1.34) (Supplementary Table 5). P-values for trend are 0.004 for CHD, 0.49 for stroke, and <0.001 for heart failure. No significant interactions were detected between the CVH trajectory groups and other covariates (age, sex, race/ethnicity, income, education, and marital status). Results from IPWs models were similar (Supplement).

**Table 2:** Multivariable-adjusted hazard ratios for incident CVD and all-cause mortality according to CVH trajectory groups.

	Adjusted Hazard Ratio (95% Confidence Interval)		
	CVD	Mortality	CVD/Mortality
High group	1 (reference)	1 (reference)	1 (reference)
Medium group	1.17 (0.86–1.59)	1.15 (0.87–1.53)	1.12 (0.89–1.41)
Persistently low group	**1.49 (1.15–1.93)**	**1.34 (1.06–1.70)**	**1.39 (1.15–1.69)**
P-value for trend	**0.002**	**0.014**	**<0.001**

## 4 Discussion

In this large multi-ethnic cohort free of prevalent CVD across four examination cycles, we identified three distinct trajectory groups of ideal CVH status. The persistently low trajectory group was estimated to constitute 45.3% of the sampled population, maintaining the lowest probability of achieving ideal CVH status over 11 years. Participants in the persistently low trajectory group had a 49% higher risk of incident CVD and 34% higher risk of death, relative to those with a persistently ideal CVH status in the high trajectory group. In addition, being in the persistently low group was associated with a 87% increase in risk of CHD and a 110% increase in risk of heart failure, but not stroke.

Our study identified a large proportion of participants falling into the persistently non-ideal CVH group and elucidated the trends of individual CVH metrics within this group. Similar to the patterns observed in the medium and high trajectory groups, the prevalence of ideal CVH metrics declined over the 11-year follow up among participants in the persistently low trajectory group, mostly from decreases in the proportions of those with ideal blood pressure, blood glucose, and cholesterol levels. Blood glucose showed a relatively high proportion of ideal levels initially but had a large drop over the follow up period, which suggests more participants’ glucose control deteriorated or became medication-dependent later in life. These findings are consistent with those reported in the whole sample of participants from the Framingham Heart study [2]. Thus, the current study emphasizes the importance of blood pressure control, lipid lowering, and glucose management in high-risk groups identified by our group-based trajectory modeling. These participants could be potential candidates for CVH lifestyle or pharmacological interventions targeted specifically at improving ideal CVH status.

Our study saw a relatively unchanged level of ideal BMI, while Framingham Heart study [2] observed a decrease in ideal BMI. This could possibly be explained by the fact that the mean age of our study is higher than the Framingham Heart Study (mean [SD] age at baseline, 60.1 [9.5] years at Exam 1 in our study vs. 55.3 [9.8] years in the Framingham Heart Study), and our participants might have had plateaued body weights as they aged.

We saw an upward trend in ideal smoking status among participants. While this reflects the success of smoking cessation as a public health priority to improve CVH, it is worth noting that more than 20% of the participants in the persistently low trajectory still had non-ideal smoking status at baseline, meaning they were either currently smoking or had quit within 12 months at the time of the exam. Since individuals in the persistently low trajectory already have less ideal status in their remaining CVH metrics, smoking would further exacerbate their already accelerated process of atherosclerosis and increase their risks of CVD and other unfavorable health outcomes. This study reemphasizes the importance of the smoking cessation campaign to improve CVH, especially among the high-risk population with nonideal CVH.

The results from our study build on previous research and provide insights into current understanding on the association of CVH trajectories and subsequent health outcomes. Two previous studies [2,3] reported on associations of changes in CVH status between two exam waves with risks of incident CVD and death. In the Framingham Heart Study [2], four groups were created describing changes in CVH score using each participant's first and last CVH scores, based on the cut-off of 8 (out of 14) to define low or high cardiovascular health on each occasion. In this study, the low-high, high-low, low-high groups were all identified to have elevated risks of CVD and mortality compared to the high-high group. However, because the time interval between first and last CVH scores among participants varied considerably, CVH changes could simply reflect the effect of the various length of the observation period instead of the natural variation within a fixed observation period for all individuals. Indeed, when van Sloten et al. [3] used data from the UK Whitehall II study to examine the changes either in dichotomized or trichotomized CVH groups between 1985/1988 and 1997/1999 in all participants with measures at both time points, there was no consistent relationship between the direction of change in the category of cardiovascular health and risk of CVD or mortality. The results from our study showed a linear trend between trajectory groups of achieving ideal CVH and risk of CVD and mortality, thus adding significantly to our understanding of complex dynamic longitudinal patterns of achieving ideal CVH status and the subsequent risk of CVD and death. Our findings are consistent with the hypothesis that not maintaining ideal CVH, especially having the lowest level of achieving ideal CVH throughout, predisposes individuals to adverse cardiovascular and health outcomes. Our study thus emphasizes the importance of maintaining ideal CVH in the primordial and primary prevention of CVD. Specifically, all three CVH trajectories in our study showed relatively mild changes of probabilities of achieving ideal CVH across the 11 years, with slight decline at the end of the 11 years in the high and medium models and no change in the persistent low model. These trajectories suggest that a given individual's CVH is unlikely to have shift drastically over a decade, and those who start with high CVH tend to remain with high CVH, and those start with low CVH tend to remain with low CVH. Given the median age of 60.1 years of our study sample at the initial exam, this study emphasizes the importance of achieving and acquiring an ideal CVH status early on in life, at least in the 50 s and 40 s.

Our study used GBTM to characterize CVH trajectories with a dichotomized measure of achieving ideal CVH. GBTM has been previously used to characterize CVH trajectories [6,7,16]. For example, Wu et al. [7] reported five CVH sub-group trajectories of the Kailuan study cohort using the continuous variable of AHA-defined CVH scores. Nonetheless, the present study extends the results of applying GBTM to characterize CVH trajectories based on the continuous scale, as used in previous studies, to a dichotomized measure. Compared to a continuous measure, a dichotomized measure (ideal vs. non-ideal), as used in our study, could be an easier measure to interpret in a clinical context.

The longitudinal trajectory modeling of the dichotomized measure of achieving ideal CVH in our study is more precise than cross-sectional measures. The dichotomized measure of ideal vs. non-ideal CVH itself is not a novel concept. Indeed, cross-sectional non-ideal CVH metrics have been well reported to be associated with various undesired cardiovascular end points [17,18] and non-cardiovascular diseases [19]. However, the longitudinal trajectory modeling of the dichotomized measure, as used in our study, adds more information than traditional cross-sectional measures of the dichotomized variable. For example, our study produced three trajectory groups, including a medium trajectory group with a moderate probability of achieving ideal CVH, which provide us with extra insights that are not found in a cross-sectional measure of the dichotomized variable. Indeed, the medium and the persistently low groups had different risks. Thus, distinguishing the persistently low group from the medium group might be clinically meaningful, as it allows us to narrow down to a smaller group with elevated risks.

We are hopeful that in the future, as more clinical data are longitudinally collected from patients and more databases are integrated with the advancement of digital health technology [20,21], larger clinical cohorts with more data points will be constructed for trajectory modeling. As such, more trajectories patterns might emerge, and risk stratification might become more precise. We would encourage further study on the predictive value and usefulness of trajectory modeling in identifying high-risk individuals, as a step-up to the single timepoint risk prediction currently used in clinics.

### 4.1 Strengths

Our study sample is a racially/ethnically diverse cohort. In addition, our study sample from MESA is inclusive of older adults (mean [SD] age at baseline at Exam 1, 60.1 [9.5] years). Thus, our results could provide additional insights into CVH trajectories and subsequent risk prediction of today's aging population.

### 4.2 Limitations

Our study is limited by the lack of a diet component in our CVH metrics. Diet is part of the original AHA-defined CVH metrics but was not included in our study due to its unavailability at Exam 2 and Exam 3. However, only 0.3% of the participants have an ideal diet score. Therefore, we do not anticipate the lack of the diet component would alter our conclusions, and if anything would only cause an underestimation of the prevalence of non-ideal CVH. Additionally, in 2022, AHA updated the construct of CVH, known as Life's Essential 8, to include sleep health as a new component of CVH in addition to the seven components mentioned previously [22]. However, sleep health is not included in our CVH metrics, as this piece of data was not measured in MESA. Regarding this lack of data, we would like to emphasize the importance of measuring diet and sleep data consistently in future cardiovascular clinical studies as they are crucial components of CVH that have been traditionally overlooked.

Our study is also potentially limited by selection bias. To address this limitation, we conducted an inverse probability weights analysis and found similar results after accounting for participants who did not attend all the exams.

## 5 Conclusions

In this multi-ethnic cohort, persistently nonideal CVH is a common trajectory. The trajectory patterns emphasize the importance of maintaining ideal CVH status early in life. Targeted cardiovascular prevention programs might provide benefits to individuals with persistently nonideal CVH given their elevated risk of subsequent CVD and mortality.

**Fig. 4:**
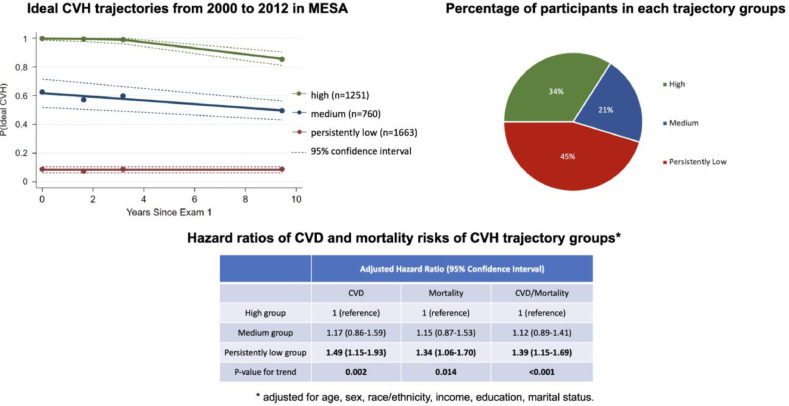
(a) Ideal CVH trajectories from Exam 1 to Exam 5. (b) Pie chart highlighting the percentage of participants in each trajectory group. (c) Table showing adjusted hazard ratios of CVD, mortality, and CVD/mortality of CVH trajectory groups.

## Statement of authorship

Qicong Sheng designed the study, conducted the analysis, and wrote the paper.

Jie Ding designed the study, conducted the analysis, and provided final review.

Yumin Gao provided final review.

Reshmi JS Patel provided final review.

Wendy S Post provided final review.

Seth S Martin designed the study and provided final review.

## Sources of funding

MESA is funded by contracts 75N92020D00001, 75N92020D00002, 75N92020D00003, 75N92020D00004, 75N92020D00005, 75N92020D00006, 75N92020D00007, N01-HC-95159, N01-HC-95160, N01-HC-95161, N01-HC-95162, N01-HC-95163, N01-HC-95164, N01-HC-95165, N01-HC-95166, N01-HC-95167, N01-HC-95168, N01-HC-95169, and HHSN268201500003I from the 10.13039/100000050National Heart, Lung, and Blood Institute, and by grants UL1-TR-000040, UL1-TR-001079, and UL1-TR-001420 from the 10.13039/100006108National Center for Advancing Translational Sciences (10.13039/100006108NCATS).

Dr. Martin has received funding/research support from the Maryland Innovation Initiative, Wallace H. Coulter Translational Research Partnership, Louis B. Thalheimer Fund, the Johns Hopkins Individualized Health Initiative, the 10.13039/100000968American Heart Association (20SFRN35380046, 20SFRN35490003, COVID19–811000, #878924, and #882415), the 10.13039/100006093Patient-Centered Outcomes Research Institute (ME-2019C1–15328), the 10.13039/100000002National Institutes of Health (P01 HL108800 and R01AG071032), the 10.13039/100015926David and June Trone Family Foundation, the Pollin Digital Innovation Fund, the PJ Schafer Cardiovascular Research Fund, Sandra and Larry Small, CASCADE FH, Apple, iHealth, 10.13039/100006785Google, and 10.13039/100002429Amgen.

Qicong Sheng has received funding from Dean's Summer Research Fund from 10.13039/100012304Johns Hopkins University School of Medicine.

## Disclosures

Dr. Martin is a founder of and holds equity in Corrie Health. Dr. Martin has received personal fees for serving on scientific advisory boards for Amgen, AstraZeneca, Dalcor, Kaneka, Novartis, Novo Nordisk, Regeneron, Sanofi, and 89bio. He is a coinventor on a system for low-density lipoprotein cholesterol estimation, which is free and open-source.

## Declaration of Competing Interest

The authors declare that they have no known competing financial interests or personal relationships that could have appeared to influence the work reported in this paper.

## Appendix Supplementary materials

Image, application 1
