# Supplementary material for: Cardiovascular health trajectories and subsequent cardiovascular disease and mortality: The multi-ethnic study of atherosclerosis (MESA)
Source: Am J Prev Cardiol. 2022 Dec 9;13:100448. doi: 10.1016/j.ajpc.2022.100448 (PMC9798133; doi:10.1016/j.ajpc.2022.100448)
Supplement: Supplementary file 1 [file mmc1.docx]

**Supplemental Material**

**Supplemental Methods**

Inverse probability weights (IPWs) to account for selective dropouts

In sensitivity analyses, we used IPWs to account for non-random censoring due to loss to follow-up or dropouts. In the first step for each participant, the IPWs were calculated by using pooled multivariable logistic regression predicting the probability of remaining free from drop out and the final IPWs were the inverse probability of remaining free from drop out. Participants last observed alive and CVD free for more than 1 year prior to December 31, 2018, were considered dropouts. The models included exposure (i.e., CVH groups), follow-up time (using restricted quadratic splines), other baseline covariates including age (spline), sex, race & education, as well as all pairwise interactions. We stabilized these final weights by multiplying them with the probability of remaining free from drop out derived from the model including only exposure (i.e., CVH groups) and follow-up time (spline). In the pooled logistic regression model, each participant contributed to up to 96 records (i.e., monthly records for eight years), and the weights were cumulatively multiplied for each participant. The estimated stabilized weights had a mean of 1.00 (with a standard deviation of 0.06) and ranged from 0.88 to 3.10. In the second step, the IP-weighted Cox model was constructed by weighting participants according to their estimated weights, with outcome of time to CVD or death, and CVH group status as the sole covariate. IPWs upweights participants with characteristics related to censoring, which creates a pseudo-population in which dropouts are not associated with subjects’ characteristics and thus reduce the impact of selection bias arising from dropouts.

**Supplementary Table 1.** Demographic characteristics of included and excluded participants at Exam 1 (2000-2002)

|  | **Included**  **(n=3674) (%)** | **Excluded**  **(n=3140) (%)** | **P-value** |
| --- | --- | --- | --- |
| **Age** (mean [sd]) | 60.1 [9.5] | 64.6 [10.5] | < 0.001 |
| **Sex** | | | |
| Women | 53.9 | 51.7 | 0.06 |
| **Race/Ethnicity** | | | |
| White | 40.5 | 36.1 | < 0.001 |
| Chinese-American | 12.8 | 10.6 |  |
| African-American | 25.2 | 30.8 |  |
| Hispanic | 21.5 | 22.5 |  |
| **Education** | | | |
| ≤ High-school graduate | 30.8 | 42.6 | < 0.001 |
| Some college or college graduate | 47.8 | 43.4 |  |
| > College graduate | 21.4 | 14.0 |  |
| **Marital Status** | | | |
| Married/Living as married | 64.2 | 56.8 | < 0.001 |
| **Income** | | | |
| < $40000 | 43.1 | 59.9 | < 0.001 |
| ≥ $40000 | 56.9 | 40.1 |  |

**Supplementary Table 2**. Definitions of the CVH metrics.

| **Components** | **Blood cholesterol** | **Blood pressure** | **Blood glucose** | **Physical activity** | **Smoking** | **BMI** |
| --- | --- | --- | --- | --- | --- | --- |
| Ideal | Total cholesterol <200 mg/dL without treatment | SBP <120 and DBP <80 mmHg without treatment | Fasting plasma glucose <100 mg/dL without treatment | ≥150 min/week moderate or ≥75 min/week vigorous or ≥150 min/week moderate and vigorous physical activities | Never smoked, or quit smoking more than 12 months ago | BMI <25 kg/m^2^ |
| Intermediate | Total cholesterol 200-239 without treatment, or <200 mg/dL with treatment | SBP 120-139 or DBP 80-89 without treatment, or SBP <120 and DBP < 80 mmHg with treatment | Fasting plasma glucose 100-125 without treatment, or <100 mg/dL with treatment | 1-149 min/week moderate or 1-74 min/week vigorous or 1-149 min/week moderate and vigorous physical activities | Smoked in the past and quit smoking less than 12 months ago | BMI 25-30 kg/m^2^ |
| Poor | Total cholesterol ≥240 mg/dL | SBP ≥140 or DBP ≥90 mmHg | Fasting plasma glucose ≥126 mg/dL | None | Currently smoking | BMI ≥30 kg/m^2^ |

**Supplementary Table 3**. Model fit statistics

| Model | BIC (N = 14696) | BIC (N = 3674) | AIC | >5% per group | Entropy |
| --- | --- | --- | --- | --- | --- |
| 2 Groups | –8130.40 | –8124.16 | –8069.22 | Yes | Yes |
| 3 Groups | –8098.34 | –8088.63 | –8045.17 | Yes | Yes |
| 4 Groups | –8107.27 | –8094.10 | –8035.12 | Yes | Yes |
| 5 Groups | –8130.15 | –8113.51 | –8039.00 | Yes | Yes |

**Supplementary Table 4.** Percentages of participants with ideal CVH metrics by ideal CVH trajectory groups

| CVH Component | % of participants in the ideal category **(high trajectory participants, n=1251)** | | | |
| --- | --- | --- | --- | --- |
|  | Exam 1 | Exam 2 | Exam 3 | Exam 5 |
| BMI | 56.4 | 56.5 | 56.3 | 52.2 |
| Blood pressure | 62.9 | 63.1 | 62.8 | 47.1 |
| Blood glucose | 94.4 | 91.8 | 92.8 | 78.6 |
| Cholesterol | 62.7 | 59.4 | 58.0 | 44.8 |
| Physical activity | 79.0 | 78.2 | 79.5 | 76.6 |
| Smoking | 95.2 | 96.1 | 96.5 | 97.2 |

| CVH Component | % of participants in the ideal category **(medium trajectory participants, n=760)** | | | |
| --- | --- | --- | --- | --- |
|  | Exam 1 | Exam 2 | Exam 3 | Exam 5 |
| BMI | 30.1 | 28.2 | 30.3 | 32.1 |
| Blood pressure | 36.4 | 35.9 | 36.3 | 25.7 |
| Blood glucose | 85.4 | 79.2 | 79.9 | 65.6 |
| Cholesterol | 45.7 | 44.2 | 41.6 | 40.2 |
| Physical activity | 63.3 | 58.8 | 62.0 | 70.1 |
| Smoking | 90.0 | 91.3 | 92.4 | 95.4 |

| CVH Component | % of participants in the ideal category **(persistently low trajectory participants, n=1663)** | | | |
| --- | --- | --- | --- | --- |
|  | Exam 1 | Exam 2 | Exam 3 | Exam 5 |
| BMI | 9.4 | 9.8 | 9.6 | 11.7 |
| Blood pressure | 19.9 | 17.7 | 17.2 | 11.6 |
| Blood glucose | 61.1 | 56.3 | 57.3 | 42.9 |
| Cholesterol | 36.4 | 34.3 | 32.2 | 27.2 |
| Physical activity | 47.1 | 43.8 | 47.0 | 48.0 |
| Smoking | 78.6 | 79.6 | 80.2 | 85.7 |

**Supplementary Table 5.** Hazard ratios of CHD, stroke, heart failure risks of CVH trajectory groups, adjusted for age, sex, race/ethnicity, income, education, marital status.

|  | **Adjusted Hazard Ratio (95% Confidence Interval)** | | |
| --- | --- | --- | --- |
|  | CHD | Stroke | Heart failure |
| High group | 1 (reference) | 1 (reference) | 1 (reference) |
| Medium group | 1.26 (0.79-1.99) | 0.88 (0.52-1.51) | 1.38 (0.73-2.61) |
| Persistently low group | **1.87 (1.28-2.71)** | 0.85 (0.54-1.34) | **2.1 (1.25-3.52)** |
| P-value for trend | **0.004** | 0.49 | **<0.001** |

**Supplementary Figure 1.** Distribution of CVH scores in the three trajectory groups at each examination (y-axis: Number of participants; x-axis: CVH score).

**
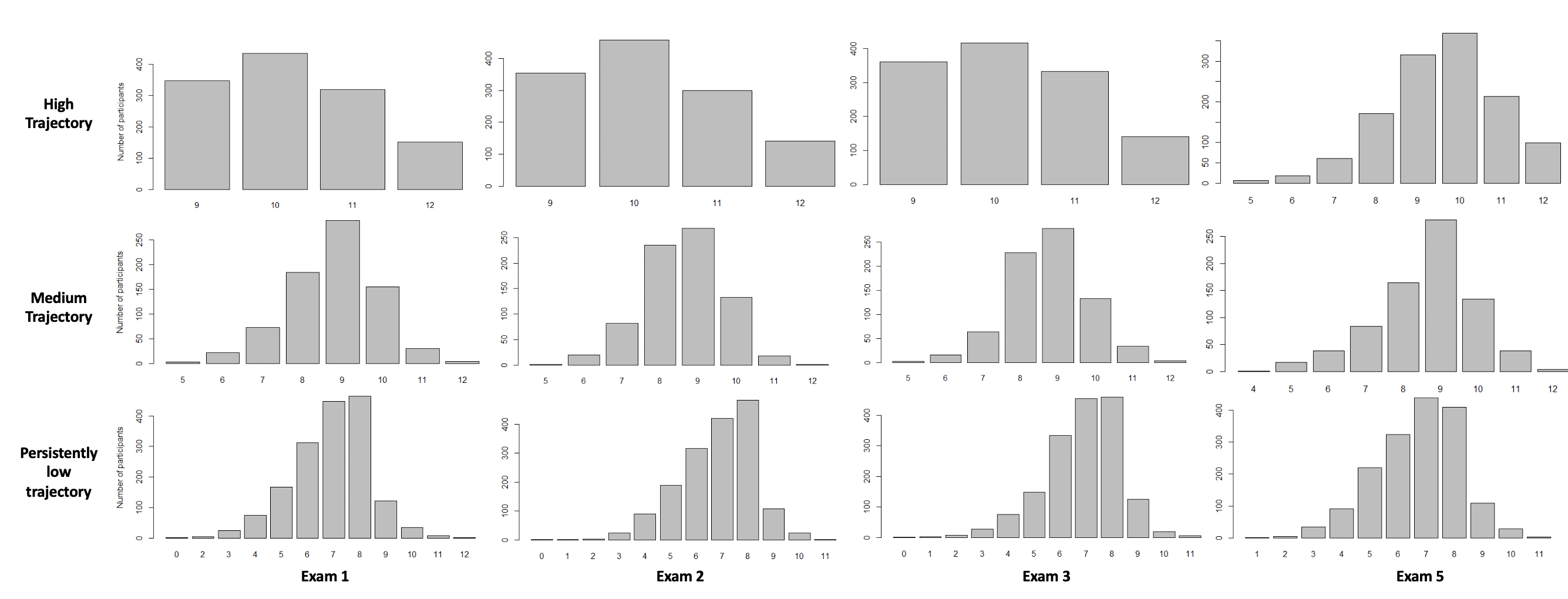
**
